# The Carbon Footprint of Bioinformatics

**DOI:** 10.1093/molbev/msac034

**Published:** 2022-02-10

**Authors:** Jason Grealey, Loïc Lannelongue, Woei-Yuh Saw, Jonathan Marten, Guillaume Méric, Sergio Ruiz-Carmona, Michael Inouye

**Affiliations:** 1 Cambridge Baker Systems Genomics Initiative, Baker Heart and Diabetes Institute, Melbourne, VIC, Australia; 2 Department of Mathematics and Statistics, La Trobe University, Melbourne, VIC, Australia; 3 Cambridge Baker Systems Genomics Initiative, Department of Public Health and Primary Care, University of Cambridge, Cambridge, United Kingdom; 4 British Heart Foundation Cardiovascular Epidemiology Unit, Department of Public Health and Primary Care, University of Cambridge, Cambridge, United Kingdom; 5 Health Data Research UK Cambridge, Wellcome Genome Campus and University of Cambridge, Cambridge, United Kingdom; 6 Department of Infectious Diseases, Central Clinical School, Monash University, Melbourne, VIC, Australia; 7 British Heart Foundation Centre of Research Excellence, University of Cambridge, Cambridge, United Kingdom; 8 The Alan Turing Institute, London, United Kingdom

**Keywords:** carbon footprint, bioinformatics, genomics, green algorithms

## Abstract

Bioinformatic research relies on large-scale computational infrastructures which have a nonzero carbon footprint but so far, no study has quantified the environmental costs of bioinformatic tools and commonly run analyses. In this work, we estimate the carbon footprint of bioinformatics (in kilograms of CO_2_ equivalent units, kgCO_2_e) using the freely available Green Algorithms calculator (www.green-algorithms.org, last accessed 2022). We assessed 1) bioinformatic approaches in genome-wide association studies (GWAS), RNA sequencing, genome assembly, metagenomics, phylogenetics, and molecular simulations, as well as 2) computation strategies, such as parallelization, CPU (central processing unit) versus GPU (graphics processing unit), cloud versus local computing infrastructure, and geography. In particular, we found that biobank-scale GWAS emitted substantial kgCO_2_e and simple software upgrades could make it greener, for example, upgrading from BOLT-LMM v1 to v2.3 reduced carbon footprint by 73%. Moreover, switching from the average data center to a more efficient one can reduce carbon footprint by approximately 34%. Memory over-allocation can also be a substantial contributor to an algorithm’s greenhouse gas emissions. The use of faster processors or greater parallelization reduces running time but can lead to greater carbon footprint. Finally, we provide guidance on how researchers can reduce power consumption and minimize kgCO_2_e. Overall, this work elucidates the carbon footprint of common analyses in bioinformatics and provides solutions which empower a move toward greener research.

## Introduction

Biological and biomedical research now requires the analysis of large and complex data sets, which would not be possible without the use of large-scale computational resources. Although bioinformatic research has enabled major advances in the understanding of a myriad of diseases such as cancers (ICGC/TCGA Pan-Cancer Analysis of Whole Genomes Consortium 2020; Kachuri et al. 2020; PCAWG Structural Variation Working Group et al. 2020) and COVID-19 (The Severe Covid-19 GWAS Group 2020), the costs of the associated computing requirements are not limited to the financial; the energy usage of computers causes greenhouse gas (GHG) emissions which themself have a detrimental impact on human health.

Energy production affects both human and planetary health. The yearly electricity usage of data centers and high-performance computing facilities (200 TWh; Jones 2018) already exceeds the consumption of countries such as Ireland or Denmark (Primary Energy Consumption by World Region 2021) and is predicted to continue to rise over the next decade (Andrae and Edler 2015; Jones 2018). Power generation, through the associated emissions of GHGs, is one of the main causes of both outdoor air pollution and climate change. Every year, it is estimated that 4.2 million deaths are caused by ambient air pollution alone, whereas 91% of the world’s population suffers from air quality below the World Health Organisation standards (Air Pollution 2016). Global warming results in further consequences on human health, economy, and society: the daily population exposure to wildfires has increased in 77% of countries (Watts et al. 2019), 133.6 billion potential work hours were lost to high temperatures in 2018 and with 220 million heatwave exposures, vulnerable populations (aged 65 years and older) are affected at an unprecedented level.

The growth of large biological databases, such as UK BioBank (Bycroft et al. 2018), All of Us Initiative (National Institutes of Health [NIH] – All of Us n.d.), and Our Future Health (Accelerating Detection of Disease – UK Research and Innovation n.d.), has substantially increased the need for computational resources to analyze these data and will continue to do so. With climate change an urgent global emergency, it is important to assess the carbon footprint of these analyses and their requisite computational tools so that environmental impacts can be minimized.

Other fields of science, such as machine learning (Strubell et al. 2019; Bender et al. 2021) and astrophysics (Jahnke et al. 2020; Portegies Zwart 2020; Stevens et al. 2020), have started to investigate the environmental impact of their computational work; this highlights the need for such study in computational biology. Notwithstanding that, alongside computation, various other aspects of biological research are responsible for substantial GHG emissions. For example, it has been estimated that powering the equipment of a typical (7–10 people) life sciences laboratory likely generates more than 20 metric tons of CO_2_e annually (Nathans and Sterling 2016). Travel also contributes to science’s carbon footprint, the carbon footprint of the annual meeting of the Society for Neuroscience (which has around 30,000 attendees) has been estimated to be approximately 22,000 metric tons CO_2_e (Nathans and Sterling 2016), roughly equivalent to the annual carbon footprint of 1,000 medium sized laboratories.

In this study, we estimate the carbon footprint of common bioinformatic tools using a model which accounts for the energy use of different hardware components and the emissions associated with electricity production. Since metrics for carbon emissions are relatively unfamiliar to most scientists, we compare the results with distances traveled by car (an average European car emits 0.175 kgCO_2_e/km; Greenhouse Gas Reporting: Conversion Factors 2019 n.d.; Helmers et al. 2019) and amounts of carbon sequestered by trees (a mature tree sequesters approximately 0.917 kgCO_2_e per month; Lannelongue et al. 2021). This study raises awareness, provides easy-to-use metrics, and makes recommendations for greener bioinformatics.

## Results

We estimated the carbon footprint of a variety of bioinformatic tools and analyses (table 1) using the Green Algorithms model and online tool (see Materials and Methods). For each software, we utilized benchmarks of running time and computational resources; in the rare cases where published benchmarks were unavailable, we used in-house analyses to estimate resource usage (see Materials and Methods). The results depend on the efficiency of the computing facility measured by its power usage effectiveness (PUE), which quantifies the additional energy the data center needs, for example, for cooling and lighting. The estimations here are based on the global average PUE of 1.67, that is, an extra 67% is necessary compared with what the servers alone demand. The global average carbon intensity (CI) (0.475 kgCO_2_e/kWh; Emissions – Global Energy & CO2 Status Report 2019 – Analysis 2019) is also used and we assume processing cores (CPU or GPU) are fully used (usage factor of 1) (see Materials and Methods).

**Table 1.** Carbon Footprint of a Range of Bioinformatic Tasks.

Task	Tool	Version	Details about the Experiments	Carbon Footprint		Tree-months	km in a Car (EU)	Running Time and Memory	Approximate Scaling (if known)
				Increase (%)	kgCO_2_e				
Genome scaffolding	SSPACE	2.0	Scaffolding 2.4 million long reads from human chromosome 14 (Hunt et al. 2014).	**—**	**0.0010**	0.0011	0.01	3 min 21 s	Linearly with number of reads.
								30 GB	
	SOAPdenovo2	r223		**+45%**	**0.0015**	0.0016	0.01	4 min 52 s	
								30 GB	
	SGA	0.9.43		**+2,752%**	**0.029**	0.032	0.17	1 h 35 min	
								30 GB	
Genome scaffolding	SSPACE	2.0	Scaffolding 23 million short reads from human chromosome 14 (Hunt et al. 2014).	**—**	**0.0027**	0.0029	0.02	8 min 40 s	
								30 GB	
	SOAPdenovo2	r223		**+34%**	**0.0036**	0.0039	0.02	1 min 38 s	
								30 GB	
	SGA	0.9.43		**+4,801%**	**0.13**	0.14	0.74	7 h 05 min	
								30 GB	
Genome assembly	Abyss	2.0	De novo assembly of a human genome from Illumina sequencing reads (Jackman et al. 2017).	**—**	**11**	12	61	20 h	
								34 GB	
	MEGAHIT	1.0.6		**+42%**	**15**	16	86	26 h	
								197 GB	
Metagenome assembly	MetaVelvet k101	1.2.01	Metagenome assembly from 100 soil samples (Vollmers et al. 2017).	**—**	**14**	16	82	1 h 06 min	
								130 GB	
	MEGAHIT	1.0.3		**+438%**	**77**	84	439	15 h 36 min	
								12 GB	
	metaSPAdes	3.8.0		**+1,206%**	**186**	203	1,065	29 h 24 min	
								60 GB	
Metagenome classification (short read)	Kraken2	2.0.7	Metagenomic classification of 5 Gb of randomly sampled reads from Zymo mock community (batch ZRC190633), containing yeast, Gram-negative, and positive bacteria (Dilthey et al. 2019)	**—**	**0.0052**	0.0057	0.03	20 min	Linearly with number of reads.
								21 GB	
	Centrifuge	1.0.4		**+141%**	**0.013**	0.014	0.07	58 min	
								12 GB	
	Kraken/Bracken	0.10.5/1.0.0		**+1,650%**	**0.092**	0.10	0.52	1 h 40 min	
								154 GB	
Metagenome classification (long read)	MetaMaps	—		**—**	**18.25**	19.91	104.27	209 h 53 min	
								262 GB	
Phylogenetics	BEAST/BEAGLE	1.8.4/2.1.2	Codon substitution modeling of extant carnivores and a pangolin group. Nucleotide substitution and phylogeographic modeling of Ebola virus genomes. See supplementary table 2, Supplementary Material online, for detailed results (Baele et al. 2019).	**—**	**0.012–0.30**	0.013–0.33	0.069–1.72	3 min 30 s to 7 h 45 min	Power law with number of loci.
								2–8 GB	
Phylogenetics	RAxml/ExaML, PhyML, IQ-TREE, FastTree	8.2.0/3.0.17, 20160530 1.4.2, 2.1.9	Over 670,000 tree inferences on about 45,000 single-gene alignments and supermatrices from 19 empirical phylogenomic data sets with thousands of genes and around 200 taxa. (Zhou et al. 2018)	**—**	**3565**	3889	20,371	300,000 h	
								8 GB	
Phylogenetics	ExaML	—	A 322-million-bp MULTIZ alignment of putatively orthologous genome regions across all species, comprising approximately 30% of an average assembled avian genome. This corresponded to the maximal orthologous sequence obtainable across all orders of *Neoaves*.(Jarvis et al. 2014)	**—**	**4372**	4769	24,983	367,920 h	
								8 GB	
RNA read alignment	HISAT2	2.0.0beta	Alignment of 10 million 100-base read pairs to Homo Sapiens hg19 genome (Baruzzo et al. 2017).	—	**0.0054**	0.0059	0.031	1 min 48 s	Linearly with number of reads.
								5 GB	
	STAR	2.5.0a		**+78%**	**0.0097**	0.011	0.055	6 min 01 s	
								35 GB	
	TopHat2	2.1.0		**+5,756%**	**0.32**	0.35	1.81	2 h 14 min	
								16 GB	
	Novoalign	3.02.13		**+17,926%**	**0.98**	1.07	5.58	32 h 12 min	
								64 GB	
RNA read alignment	HISAT2	2.0.0beta	Alignment of 10 million 100-base read pairs to Plasmodium falciparum genome (Baruzzo et al. 2017).	**—**	**0.0052**	0.0057	0.030	1 min 44 s	
								1 GB	
	TopHat2	2.1.0		**+4,519%**	**0.24**	0.26	1.37	1 h 25 min	
								13 GB	
	STAR	2.5.0a		**+7,025%**	**0.37**	0.40	2.11	2 h 27 min	
								8 GB	
	Novoalign	3.02.13		**+12,847%**	**0.67**	0.73	3.83	38 h 04 min	
								21 GB	
RNA-seq QC pipeline	FastQC, TrimGalore, bbmap/clumpify, and STAR	-/v0.6.0/-/v2.7.0e	Quality control analysis of raw reads quality of 392 samples from the Childhood Asthma Study (in-house).	**—**	**54.97**	59.97	314.11	485 h 12 min	
								8 GB	
Transcript isoform abundance estimation	Sailfish 1 core	0.6.3	Transcript isoform quantification of 100 million in silico reads generated from Flux Simulator with hg19 genome and GENCODE v19 annotation set (Kanitz et al. 2015)	**—**	**0.0081**	0.0088	0.046	42 min	Linearly with the number of reads.
								7 GB	
	Sailfish 16 cores			**+344%**	**0.036**	0.039	0.21	14 min	
								7 GB	
	Cufflinks 1 core	2.1.1		**+451%**	**0.045**	0.049	0.26	3 h 30 min	
								11 GB	
	Cufflinks 16 cores			**+3,262%**	**0.27**	0.30	1.56	1 h 45 min	
								12 GB	
	RSEM 1 core	1.2.18		**+6,982%**	**0.57**	0.63	3.28	47 h 10 min	
								9 GB	
	RSEM 16 cores			**+17,162%**	**1.40**	1.53	8.00	8 h 50 min	
								21 GB	
GWAS	Bolt-LMM	2.3	Analyses of a single trait in UK Biobank (*N* = 500,000) (Loh et al. 2018)	**—**	**4.70**	5.13	26.87	60 h 58 min	Linearly with number of variants.
								100 GB	
	Bolt-LMM	1.0		**+268%**	**17.29**	18.86	98.81	224 h 10 min	
								100 GB	
Cohort scale eQTL analysis	TensorQTL	1.0.2	Cis-eQTL mapping of 10.7 M SNPs against 18,373 genetic features in a cohort of 2,745 individuals (in-house).	**—**	**2.04**	2.22	11.7	1 h 14 min	Nonlinearly with the number of traits or the sample size.
								192 GB	
	LIMIX	2.0.3		**+9,256%**	**190.73**	208.07	1,089.9	9,705 h	
								41–221 GB	
Single cis-eQTL gene mapping	TensorQTL	—	Cis-eQTL mapping one gene from skeletal muscle in GTEx (v6p) (Taylor-Weiner et al. 2019).	**—**	**0.00001**	0.00001	0.00004	0.11 s	
								52 GB	
	FastQTL	—		**+2,681%**	**0.0002**	0.0002	0.001	30 s	
								52 GB	
Molecular dynamics simulation	AMBER	18	Simulation of a Satellite Tobacco Mosaic Virus with 1,066,628 atoms for 100 ns^a^ (NAMD Performance n.d.; The Pmemd.Cuda GPU Implementation n.d.).	**—**	**18**	19	102	75 h	
								(^b^)	
	NAMD	2.13		**+433%**	**95**	104	544	400 h	
								(^b^)	
Molecular docking	Glide	57111	Molecular docking of four DUD systems, scaled to 1 m ligands (Ruiz-Carmona et al. 2014)	**—**	**13**	14	74	1,027 h 47 min	
								0.05 GB	
	rDock	—		**+1,092%**	**154**	168	878	12,250 h	
								0.05 GB	
	AutoDock Vina	—		**+3,886%**	**514**	561	2,938	40,972 h	
								0.05 GB	

Note.—Further details for each task are included in supplementary additional file 1, Supplementary Material online.

a Note different simulation parameters between the two: AMBER18 (4fs timestep, 9 A cut-off) NAMD (2fs timestep with rigid bonds, 12 A cut-off with PME every two steps).

b No memory included due to a lack of information.

We considered a wide range of bioinformatic analyses: genome assembly, metagenomics, phylogenetics, RNA sequencing (RNAseq), genome-wide association analysis, molecular simulations, and virtual screening. We also show that choices of hardware substantially affect the carbon footprint of a given analysis, in particular cloud versus local computing platforms, memory usage, processor options, and parallel computing. The same applies to software choices, including software versions. These results present orders of magnitude and we note how the estimations are likely to scale with different parameters (e.g., sample size or number of features), but for precise estimations of specific analysis, scientists should estimate their own footprint, for example using the Green Algorithms tool (www.green-algorithms.org, last accessed 2022).

### Genome Assembly

Genome assembly is the process of combining sequencing reads (short or long reads, or a combination) into a single or a set consensus sequences for an organism. Hunt et al. (2014) compared SSPACE (Boetzer et al. 2011), SGA (Simpson and Durbin 2012), and SOAPdenovo2 (Luo et al. 2012) for genome scaffolding using contigs produced with the Velvet assembler (Zerbino and Birney 2008) and the human chromosome 14 GAGE data set (Salzberg et al. 2012); two read sets were compared, one using 22.7 million short reads (fragment length of 3 kb) and the other 2.4 million long reads (35 kb). Scaffolding the short or long reads resulted in similarly low carbon footprints (0.0010 to 0.13 kgCO_2_e) (table 1). However, SGA had a carbon footprint up to 49 times higher than the other tools (table 1), but it may be a result of the increased time needed to build the FM-index (full-text minute-space index) (Simpson and Durbin 2012). As the running time of many genome assembly tools scale linearly with the number of reads (Sutton et al. 2019), these results equate to between 0.00012 to 0.0057 kgCO_2_e (0.00013 to 0.0063 tree-months) per million short reads assembled and 0.00043 to 0.012 kgCO_2_e (0.00047 to 0.013 tree-months) per million long reads assembled. On an average, long read assembly had a carbon footprint per million reads 3.2x larger than short-read assembly for the tools we measured. All three methods had similar performance on these read sets with SOAPdenovo2 slightly outperforming SGA and SSPACE.

For whole genome assembly of humans, ABySS (Jackman et al. 2017) and MEGAHIT (Li et al. 2016) were benchmarked by Jackman et al. (2017) using Illumina short read sequencing (815 M reads, 379 M uniquely mapped reads, 6 kb mean insert size) (table 1). We estimated the carbon footprint of these tasks to be between 11 and 15 kgCO_2_e (12 to 16 tree-months), or per million reads, between 0.013 and 0.019 kgCO_2_e (0.014–0.020 tree-months). It is difficult to succinctly quantify the accuracy of these tools as it has been shown to vary greatly between use cases and data sets (Bradnam et al. 2013). Instead, relevant published benchmarks, such as Bradnam et al. (2013), Lischer and Shimizu (2017), and Jackman et al. (2017) can indicate the assembler that excels in the area of interest, for example, number of error-free bases, coverage, or continuity.

### Metagenomics

Metagenomics is the sequencing and analysis of all genetic material in a sample. Based on a benchmark by Vollmers et al. (2017), we estimated the carbon footprint of metagenome assembly with three commonly used assemblers, metaSPAdes (Nurk et al. 2017), MEGAHIT (Li et al. 2016), and MetaVelvet (k-mer length 101 bp) (Namiki et al. 2012) on 100 samples from forest soil (33 M reads, median length 360 bp). It ranged between 14 and 186 kgCO_2_e (table 1), corresponding to 0.14 to 1.9 kgCO_2_e per sample (0.2–2 tree-months). MetaSPAdes had the greatest carbon footprint but also the best performance followed by MetaVelvet and MEGAHIT, respectively.

For metagenomic classifiers, Dilthey et al. (2019) benchmarked MetaMaps (Dilthey et al. 2019), Kraken2 (Wood et al. 2019), Kraken/Bracken (Wood and Salzberg 2014; Lu et al. 2017), and Centrifuge (Kim et al. 2016). They compared these tools on approximately 5 Gb of randomly sampled reads from an Oxford Nanopore GridION sequencing run from Zymo mock communities, which comprises five Gram-positive bacteria, three Gram-negative bacteria and two types of yeast. Carbon footprints differed by several orders of magnitude, 18.25 kgCO_2_e for the long-read classifier MetaMaps but less than 0.1 kgCO_2_e for the short-read classifiers (table 1). The carbon footprints per Gb of classified reads ranged from 0.001 to 0.018 kgCO_2_e (0.001 to 0.02 tree-months) using the short-read classifiers (Kraken2, Centrifuge, Kraken/Bracken) and 3.65 kgCO_2_e (4 tree-months) when using MetaMaps. Kraken2 had the highest performance over all taxonomic ranks when all reads were assembled, followed by Kraken/Bracken, Centrifuge, and MetaMaps. However, when considering long reads (>1,000 bp), MetaMaps had the highest precision and recall for all available taxonomic levels, followed by Kraken2, Kraken/Bracken, and Centrifuge.

### Phylogenetics

Phylogenetics is the use of genetic information to analyze the evolutionary history and relationships among individuals or groups. Baele et al. (2019) benchmarked nucleotide substitution models with and without spatial location information to study the evolution of the Ebola virus during the 2013–2016 West African epidemics (1,610 genomes, 18,992 nucleotides; Dudas et al. 2017). These nucleotide substitution models are based on a four-partition model (one for each codon position and one for the intergenic region), and generalized linear models (Dudas et al. 2017) when including spatial information in the phylogeographic analysis. Additionally, Baele et al. benchmarked more complex Goldman and Yang’s (1994) codon substitution models on a set of mitochondrial genome from extant carnivores and a pangolin outgroup. For all these tasks, they utilized the Bayesian inference framework implemented in BEAST (Drummond et al. 2012) combined with BEAGLE (Ayres et al. 2012) for computational speedup.

**Fig. 1.**
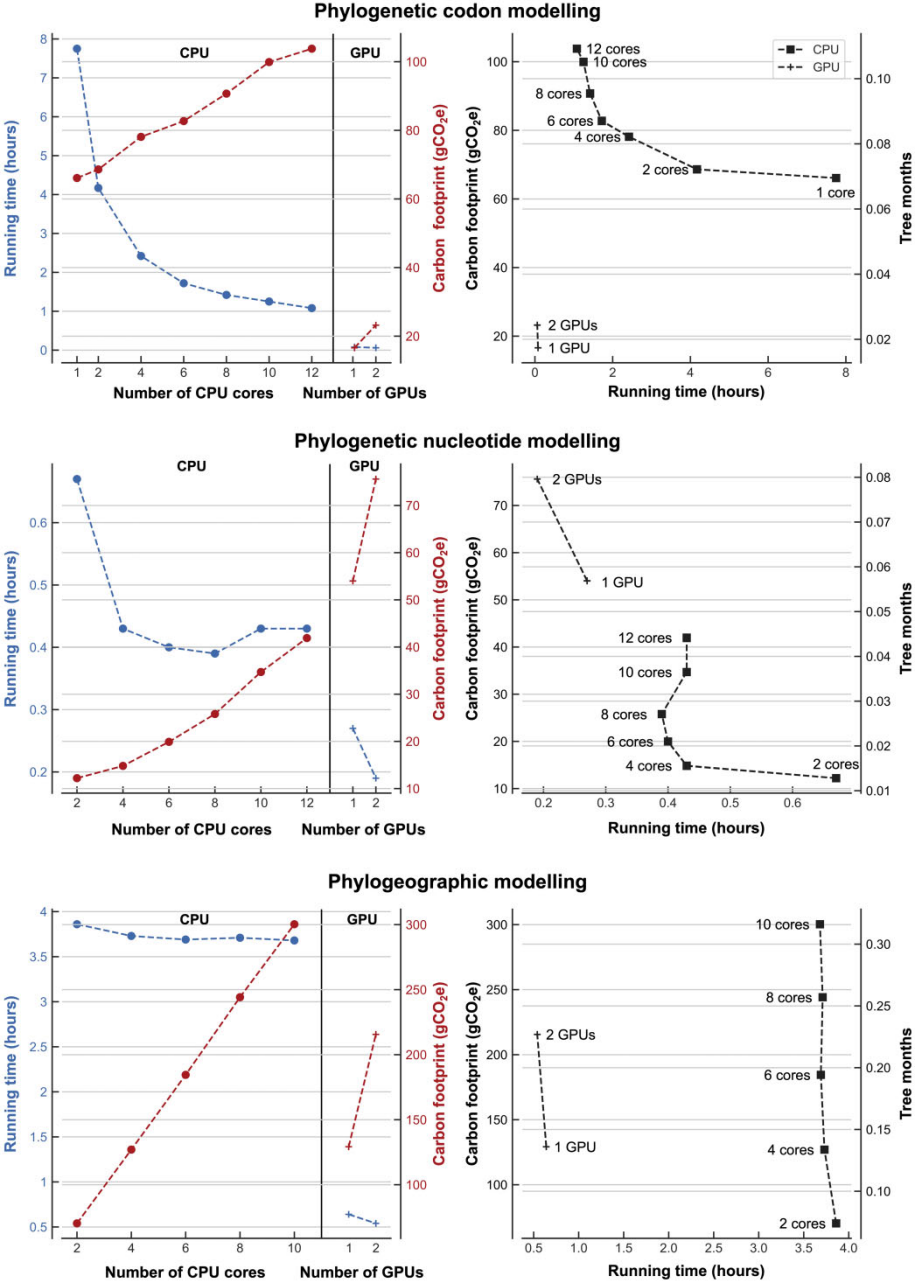
The effect of hardware choices and parallelization on carbon footprint. The carbon footprint of BEAST/Beagle implemented on multicore CPU or GPUs for three different tasks. The plots on the left detail both the running time and carbon footprint against the number of cores utilized. The plots on the right detail the running time solely against carbon footprint (contextualized with tree-months) for both CPUs and GPUs. The numerical data are available in supplementary table 2, Supplementary Material online.

We estimated the carbon footprint of nucleotide-based modeling of the Ebola virus data set was between 0.012 and 0.076 kgCO_2_e depending on hardware choices and up to 25 times higher (up to 0.30 kgCO_2_e) when including spatial information. More complex codon modeling of extant carnivores and pangolins resulted in a greater footprint, from 0.017 to 0.10 kgCO_2_e (fig. 1, table 1, and supplementary table 2, Supplementary Material online). The impact of hardware choices illustrates a trade-off between running time and carbon footprints, and is discussed in more detail below (see Parallelization and Processors). It should be noted that the running time of BEAST, and therefore its carbon footprint, scales as a power law, that is, not linearly, with the number of loci (Ogilvie et al. 2016).

We also estimated the carbon footprint of two large-scale empirical phylogenetic studies that each used over 300,000 CPU hours (table 1) (Jarvis et al. 2014; Zhou et al. 2018). As both studies were lacking hardware information, we assumed a CPU power draw of 12 W per core (the average from our database). Four different maximum likelihood-based phylogenetic programs were evaluated—RAxML (Stamatakis 2014) with ExaML (Kozlov et al. 2015), PhyML (Guindon and Gascuel 2003; Guindon et al. 2010), IQ-TREE (Nguyen et al. 2015), and FastTree (Price et al. 2010)—by conducting more than 670,000 tree inferences on 19 empirical phylogenomic data sets with thousands of genes and around 200 taxa. We estimated this would have a carbon footprint of 3,565 kgCO_2_e (3,889 tree-months or 324 tree-years). Additionally, using the maximum likelihood program ExaML, Jarvis et al. (2014) performed a 322-million-bp MULTIZ alignment of putatively orthologous genome regions across 48 species of *Neoaves* and had a similarly large carbon footprint of 4,372 kgCO_2_e (4,769 tree-months).

### RNA Sequencing

RNA sequencing is the sequencing and analysis of all RNA in a sample. We first assessed the read alignment step in RNAseq using an extensive benchmarking where Baruzzo et al. (2017) looked at different data sets of 10 million 100-base paired-end strand-specific simulated reads of two different genomes, *Homo sapiens* (hg19) and *Plasmodium falciparum* (Baruzzo et al. 2017), which have substantially differing levels of complexity (*P. falciparum* has higher rates of polymorphisms and errors). We estimated the carbon footprint of aligning two sets of reads, one to each genome (T1 human and T3 Malaria). The three most-cited software tested, STAR (Dobin et al. 2013), HISAT2 (Kim et al. 2019, 2), and TopHat2 (Kim et al. 2013), all had low recall when aligning the malaria reads to the *P. falciparum* genome, so we also assessed Novoalign (NovoAlign | Novocraft n.d.) as it performed significantly better for this task (table 1). The carbon footprints ranged from 0.0052 to 0.67 kgCO_2_e for *P. falciparum*, with Novoalign having both the best performances and the largest carbon footprint. For human read alignment, despite all four methods obtaining high recall, their footprints varied by over two orders of magnitude (0.0054 to 0.98 kgCO_2_e). As alignment tools are often reported with alignment speed (number of reads aligned in a given time) (Dobin et al. 2013; Kim et al. 2019, 2), the carbon footprints of the analyses above scale accordingly and ranged from 0.001 to 0.1 kgCO_2_e (0.001 to 0.1 tree-months) per million human or *P. falciparum* reads.

To quantify the carbon footprint of a full quality control pipeline with FastQC, we utilized 392 RNAseq read sets obtained from PBMC samples (Kusel et al. 2006, 2007), with a median depth of 45 million paired-end reads and average length 146 bp. Adapters were trimmed with TrimGalore(Babraham Bioinformatics – Trim Galore! n.d.), followed by the removal of optical duplicates using bbmap/clumpify (BBMap Guide n.d.). Reads were then aligned to the human genome reference (Ensemble GRCh 38.98) using STAR (Dobin et al. 2013). We estimated the carbon footprint of this pipeline to be 54.97 kgCO_2_e for the full data set, or 1.22 kgCO_2_e per million reads (table 1), which scales linearly with the number of reads (supplementary additional file 2, Supplementary Material online).

For transcript isoform abundance estimation, we assessed Sailfish (Patro et al. 2014), RSEM (Li and Dewey 2011), and Cufflinks (Trapnell et al. 2010) using the benchmark from Kanitz et al. (2015) on simulated human RNA-seq data (hg19). The Flux Simulator software (Griebel et al. 2012) and GENCODE (Harrow et al. 2012) were used to generate 100 million single-end 50-bp reads. The carbon footprints of this task were between 0.0081 and 1.40 kgCO_2_e (table 1), and the authors showed that the time complexity, and therefore the carbon footprint, is proportional to the number of reads. Additionally, these tools offer the option of parallelization, which can reduce running time but in this case, not carbon footprint; indeed, the decrease in running time when using 16 cores instead of one was not sufficient to offset the increase in power consumption, which resulted in a 2- to 6-fold increase in carbon footprint when utilizing 16 cores (table 1). There were significant differences between tools despite RSEM and Sailfish having similar accuracy performances in this benchmark. Since Sailfish does not perform a read alignment step and was on an average 53 times faster than RSEM, its carbon footprint was 71 times less than RSEM’s when using 1 core and 39 times less with 16 cores. Lastly, although Cufflinks is largely used for abundance estimation, its main purpose is transcript isoform assembly, resulting in a significantly lower accuracy here (at a higher carbon cost).

### Genome-Wide Association Analysis

Genome-wide association analysis aims to identify genetic variants across the genome associated with a phenotype. Here, we assessed both genome-wide association studies (GWAS) and expression qualitative trait locus (eQTL) mapping. We estimated the carbon footprint of GWAS with two different versions of Bolt-LMM (Loh et al. 2018) on the UK BioBank (Bycroft et al. 2018) (500k individuals, 93 M imputed SNPs). We found that a single trait GWAS would emit 17.29 kgCO_2_e with Bolt-LMM v1 and 4.70 kgCO_2_e with Bolt-LMM v2.3 (table 1), a reduction of 73%. GWAS typically assess multiple phenotypes, for example, metabolomics GWAS consider from several hundreds to several thousands of metabolites; since the association models in GWAS are typically fit on a per-trait basis, the carbon footprint is proportional to the number of traits analyzed. Bolt-LMM’s carbon footprint also scales linearly with the number of genetic variants (BOLT-LMM v2.3.4 User Manual 2019), meaning that a single biobank-scale GWAS using UK Biobank (500k individuals) has a carbon footprint of 0.05 kgCO_2_e per million variants (0.06 tree-months) with Bolt-LMM v2.3 and 0.2 kgCO_2_e per million variants (0.2 tree-months) with Bolt-LMM v1. However, Bolt-LMM does not scale linearly with the number of samples (*time ∼ O(N^1.5^)*; BOLT-LMM v2.3.4 User Manual 2019), which must be taken into account when scaling the values to a different sample size.

For cis-eQTL mapping, we compared the carbon footprint using either CPUs or GPUs on two data sets, first on a small sample size using skeletal muscle data from GTEx (GTEx Consortium 2017) (1 gene, 700 individuals) with a benchmark of FastQTL (CPU) (Ongen et al. 2016) and TensorQTL (GPU) (Taylor-Weiner et al. 2019; Broadinstitute/Tensorqtl (2018) 2020) from Taylor-Weiner et al. (2019). Besides, both tools were shown to yield similar mappings. Secondly, we used an in-house assessment (see Materials and Methods), to estimate the carbon footprint of a CPU-based analysis with LIMIX (Lippert et al. 2014) and with the GPU-based TensorQTL, using a larger cohort of 2,745 individuals with 18k genetic features and 10.7 m SNPs (table 1). In both cases, footprints were lower (28x and 94x) when using GPUs instead of CPUs. The scaling of eQTLs is complex, and the carbon footprint does not scale linearly with the number of traits or sample size (Lippert et al. 2014; Taylor-Weiner et al. 2019).

### Molecular Simulations and Virtual Screening

Molecular simulations and virtual screening use computational simulations to model and understand molecular behavior and in silico scanning of small molecules for drug discovery. We estimated the carbon footprint of simulating molecular dynamics of the Satellite Tobacco Mosaic Virus (1,066,628 atoms) for 100 ns (nanoseconds) using AMBER and NAMD (NAMD Performance n.d.; The Pmemd.Cuda GPU Implementation n.d.) (Case et al. 2005; Phillips et al. 2005) and obtained between 18 and 95 kgCO_2_e, which corresponds to 0.2 to 1 kgCO_2_e per ns (table 1). It should be noted that there are small discrepancies between the simulation parameters used by the tools so they cannot be compared directly (table 1), and due to a lack of information, neither of these estimations include the power usage from memory.

Using a benchmark from Ruiz-Carmona et al. (2014), we estimated the carbon footprint of three molecular docking methods, AutoDock Vina, Glide, and rDock (Friesner et al. 2004; Trott and Olson 2010; Ruiz-Carmona et al. 2014). The data originate from four systems (ADA, COMT, PARP, and Trypsin) from the Directory of Useful Decoys benchmark set (Huang et al. 2006). To estimate their carbon footprints, we used the average computational running times for a 1 million ligand campaign and found values ranging from 13 to 514 kgCO_2_e (table 1). Glide was the fastest tool and had the smallest footprint, although it is not freely available. Of the two freely available tools (AutoDock Vina and rDock), rDock had the smallest carbon footprint with a performance comparable to Glide (Ruiz-Carmona et al. 2014).

### Local versus Cloud Data Center, and the Role of Geography

Cloud computing facilities and large data centers are optimized to significantly reduce overhead power consumption such as cooling and lighting, and as such are often more energy efficient than smaller facilities. A report from 2016 estimated for example that energy usage by data centers in the United States could be reduced by 25% if 80% of the smaller data centers were aggregated into larger and more efficient data centers (hyperscale facilities) (Shehabi et al. 2016). Compared with the global average PUE of 1.67, Google Cloud’s average PUE of 1.11 (Efficiency – Data Centers – Google n.d.) reduces the carbon footprint of a task by 34%. Other cloud providers also achieve low PUEs, Microsoft Azure reduces the carbon footprint by 33% (PUE = 1.125; Microsoft 2015) and Amazon Web Service by 28% (PUE = 1.2; AWS & Sustainability n.d.).

**Fig. 2.**
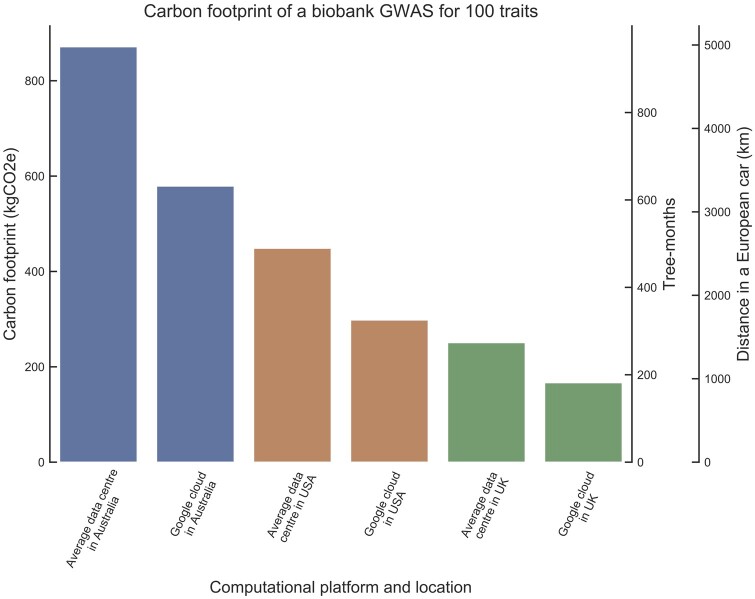
Impact of location and computational platform on carbon footprint. Carbon footprint (in kgCO_2_e, tree-months, and European car km) of a biobank scale 100 trait GWAS in various locations and platforms. Average data centers have a PUE of 1.67 (Andy 2019), Google cloud has a PUE of 1.11 (Efficiency – Data Centers – Google n.d.), Australia has a CI of 0.88 kgCO_2_e/kWh, the United States 0.453 kgCO_2_e/kWh, and the UK 0.253 kgCO_2_e/kWh (Carbonfootprint.Com – International Electricity Factors 2020).

The use of cloud facilities may also enable further reductions of carbon footprint by allowing users to choose a geographic location with relatively low CI. As an example, we found that a typical GWAS of UK Biobank considering 100 traits using the aforementioned GWAS framework (see Genome-Wide Association Analysis) together with BoltLMM v2.3 on a Google Cloud server in the UK would lower the carbon footprint by 81% when compared with the average local data center in Australia (fig. 2), potentially saving 705 kgCO_2_e (769 tree-months, or 64 tree-years). To find the optimal strategy for specific analysis and facilities, it is best to directly use the Green Algorithm calculator (www.green-algorithms.org, last accessed 2022).

### Parallelization

It is common practice to use parallelization to share the workload between several computing cores and reduce the total running time. However, it has been shown that this can increase carbon footprint (Lannelongue et al. 2021) and we found that parallelization frequently results in trade-offs between running time and carbon footprint. A general optimal solution to this trade-off is difficult to find as the relationship between carbon footprint and number of cores used may not be linear depending on the power management strategy of the servers. For modeling purposes, we assume here that cores are allocated independently to different users and that each core is used at 100%.

In some cases, the reduction in running time is substantial. For example, executing the phylogenetic codon model (see Phylogenetics) on a single core would take 7.8 h and emit 0.066 kgCO_2_e, but with two cores, the carbon footprint increased by only 4% while running time was decreased by 46% (1.9x speedup) (fig. 1 and supplementary table 2, Supplementary Material online). With 12 cores, running time decreased 86% (7.2x speedup) but the carbon footprint increased by 57%. In other cases, speedup was marginal, making the added GHG emissions unnecessary. For example, the phylogeographic model had a running time of 3.86 h with a carbon footprint of 0.070 kgCO_2_e when using two cores; increasing to ten cores reduced running time by only 5% but increased carbon footprint by 4-fold (fig. 1 and supplementary table 2, Supplementary Material online).

### The Impact of Memory

**Fig. 3.**
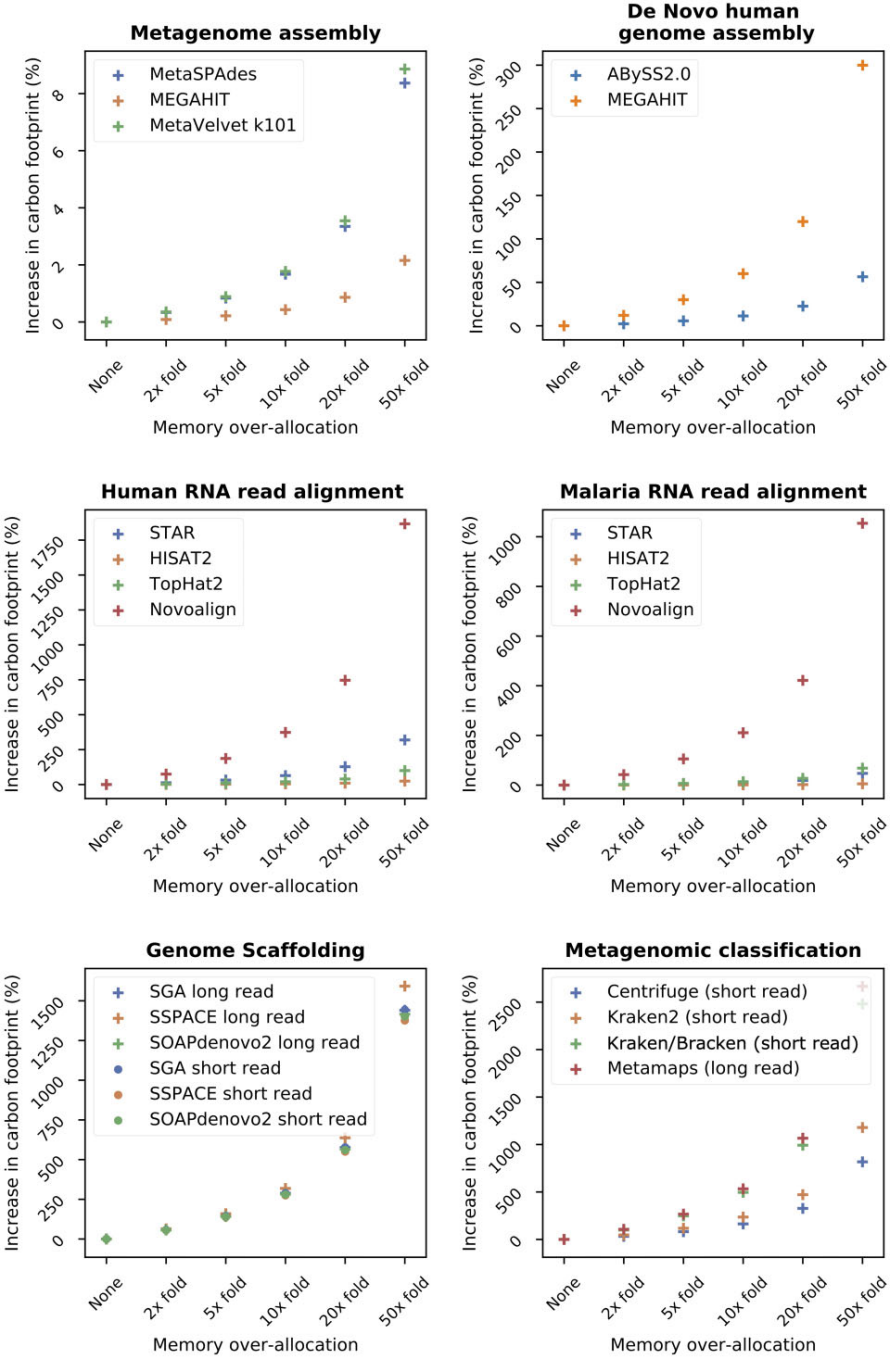
Over-allocating memory increases a given algorithm’s carbon footprint. We modeled how over-allocating the memory for a given algorithm increases its carbon footprint and this effect is increased for algorithms with larger memory requirements. Each plot details the percentage increase in carbon footprint as a function of memory overestimation for a variety of bioinformatic tools and tasks. The numerical data are available in supplementary table 1, Supplementary Material online.

Provided memory is mobilized and not idle, its power consumption depends mainly on the memory available, not on the memory used (Karyakin and Salem 2017; Lannelongue et al. 2021). Thus, having too much memory available for a task results in unnecessary energy usage and GHG emissions. Although memory is usually a fixed parameter when working with a desktop computer or a laptop, on most computational servers and cloud platforms, the user can choose the memory allocated. Given it is common practice to over-allocate memory out of caution, we modeled the impact of memory allocation on carbon footprint in bioinformatics (fig. 3 and supplementary table 1, Supplementary Material online).

We showed that, while increasing the allocated memory always increases the carbon footprint, the effect is particularly significant for tasks with large memory requirements (fig. 3 and supplementary table 1, Supplementary Material online). For example, in de novo human genome assembly, MEGAHIT had higher memory requirements than ABySS (6% vs. 1% of total energy consumption); as a result, a 5-fold over-allocation of memory increases carbon footprint by 30% for MEGAHIT and 6% for ABySS. Similarly, in human RNA read alignment (fig. 3 and supplementary table 1, Supplementary Material online), Novoalign had the highest memory requirements (37% of its total energy vs. less than 7% for STAR, HISAT2, and TopHat2) and a 5x over-allocation in memory would increase its footprint by 187% compared with 32% for STAR, 2% for HISAT2, and 10% for TopHat2.

### Processors

We estimated the carbon footprint of algorithms executed on both GPUs and CPUs. For cis-eQTL mapping (see Genome-Wide Association Analysis), we estimated that, compared with CPU-based FastQTL and LIMIX, using a GPU-based software like TensorQTL can reduce the carbon footprint by 96% and 99% and the running time by 99.63% and 99.99%, respectively (table 1). For the codon modeling benchmark (see Phylogenetics), utilizing GPUs had a speedup factor of 93x and 13x when compared with 1 and 12 CPU cores, resulting in a decrease in carbon footprint of 75% and 84% respectively. These estimations demonstrate that GPUs can be well suited to both reducing running time and carbon footprint for algorithms.

However, there are situations where the use of GPUs can increase carbon footprint. Using a GPU for phylogenetic nucleotide modeling (see Phylogenetics), instead of 8 CPU cores, decreased running time by 31% but also doubled the carbon footprint. We estimated that a single GPU would need to run the model in under 4 min to match the CPU’s carbon footprint, as opposed to the 16 min it currently takes. Similarly, using a GPU for the phylogeographic modeling of the Ebola virus data set (see Phylogenetics) reduced the running time by 83% (6x speedup) when compared with the method with the lowest footprint (2 CPU cores) but increased carbon footprint by 84%. The equations used for this estimation are in supplementary note 1, Supplementary Material online, but a simple approximation can be used by scaling the running time of the GPU by the ratio of the power draws of the CPU and GPU. For example, we compared the popular Xeon E5-2683 CPU (using all 16 cores, power draw of 120 W) to the Tesla V100 GPU (300 W) and found that, to have the same carbon footprint with both configurations, an algorithm needs to run approximately 2.5 times (300/120) faster on GPU than CPU.

## Discussion

In this work, we estimated the carbon footprint of various bioinformatic algorithms. Additionally, we investigated how memory over-allocation, processor choice, and parallelization affect carbon footprints, and showed the impact of transferring computations to cloud facilities.

This study made a series of important findings:

1. For the same task, there can be orders of magnitude differences between the carbon footprints of the tools available, despite similar performances. This highlights the importance of factoring in GHG emissions when choosing a software.
2. Limiting parallelization can reduce carbon footprints. Especially when the running time reduction is marginal, the carbon cost of parallelization should be closely examined. Besides, such methods to obtain faster running times may encourage scientists to run more computations; this rebound effect can increase carbon footprints further.
3. Despite being often faster, GPUs do not necessarily have a smaller carbon footprint than CPUs, and it is useful to assess whether the running time reduction is large enough to offset the additional power consumption. In particular, when new hardware needs to be acquired, the environmental impact of manufacturing it should be taken into account.
4. Using energy-efficient data centers, either local or cloud-based, can reduce carbon footprints by approximately 34% on an average.
5. Substantial reductions in carbon footprint can be made by performing computations in energy-efficient countries with low CI.
6. Carbon offsetting, which consists of supporting GHG-reducing projects, can be a way to balance the GHG emissions of computations. Although a number of cloud providers take part in this (AWS & Sustainability n.d.; Google Cloud Environment | Go Green n.d.; Global Infrastructure | Microsoft Azure n.d.), the real impact of carbon offsetting is debated and reducing the amount of GHG emitted in the first place should be prioritized.
7. Over-allocating memory resources can unnecessarily, and significantly, increase the carbon footprint of a task, particularly if this task has high memory usage already. To decrease energy waste, one should allocate memory in a mindful manner and mobilize the minimum amount of memory needed for the task, while being careful not to under allocate memory either, as failed jobs are another source of energy waste. The modeling of the impact of overallocation here is based on a number of assumptions regarding memory power draw (Desrochers et al. 2016; Karyakin and Salem 2017) and orders of magnitude rather than exact values should be remembered. Additionally, software could be optimized to minimize memory requirements, potentially moving some aspects to disk where energy usage is far lower. However, this introduces a trade-off between memory usage and running time, and developers need to identify the most sustainable option on a case-by-case basis.
8. A simple way to reduce the carbon footprint of a given algorithm is to use the most up to date software. We showed that updating a common GWAS software reduced carbon footprint by 73%, indicating that this may be the quickest, easiest, and potentially most impactful way to reduce one’s carbon footprint.

There are a number of assumptions made when estimating the energy usage and carbon footprint of a given algorithm. These assumptions, and the associated limitations, have been discussed in detail within Lannelongue et al. (2021). In particular, we had to assume that processors were fully used (usage factor of 1) during the task, which is likely to slightly overestimate energy usage. Another noteworthy limitation of the work here is that many of the carbon footprints estimated are for a single run of any given tool; however, most algorithms have parameters that must be fine-tuned through trial and error, frequently extensively so. For example, in GWAS, various adjustments are made to the initial association analysis to reduce nonbiological variation, such as different phenotype normalizations, batch-effect correction, and ancestry-effect adjustments. Each of these adjustments multiplies the analysis’ total carbon footprint and therefore the real GHG emissions are likely to be orders of magnitude greater than reported here.

There are other areas of computational biology, such as imaging or artificial intelligence analyses, that are not estimated here but are likely have substantial carbon footprints. Similarly, there are a number of other popular bioinformatics algorithms that have not been estimated within this study, examples include BLAST (Altschul et al. 1990), GROMACS (Spoel et al. 2005), and GATK (McKenna et al. 2010). Finally, it is generally the case that at least some parameters needed to estimate the carbon footprint are missing from published articles, for example, running time, hardware information, or software versions. If we are to fully understand the carbon footprint of the field of bioinformatics, or any computational research, it is crucial that this information is reported systematically (processor running time, memory usage, hardware, and software information) and that authors estimate their own carbon footprint using reliable tools.

This study is, to the best of our knowledge, the first to estimate the carbon footprint of common bioinformatics tools. We also investigated how parallelization, memory over-allocation, and hardware choices affect GHG emissions and showed that they could be reduced by utilizing efficient computing facilities. Finally, we outlined a range of ways bioinformaticians can use to may their carbon footprint.

## Materials and Methods

### Selection of Bioinformatic Tools

We estimated the carbon footprint of a range of tasks across the field of bioinformatics: genome and metagenome assembly, long and short reads metagenomic classification, RNA-seq and phylogenetic analyses, GWAS, eQTL mapping algorithms, molecular simulations, and molecular docking (table 1). For each task, we curated the published literature to identify peer-reviewed studies which computationally benchmarked popular tools. To be selected, publications had to report at least the running time and preferably memory usage and hardware used for the experiments, in particular the model and number of processing cores. We selected ten publications for this study (table 1). Besides, as we could not find suitable benchmarks to estimate the carbon footprint of cohort-scale eQTL mapping and RNA-seq quality control pipelines, we estimated the carbon footprint of these tasks using in-house computations. These computations were run on the Baker Heart and Diabetes Institute’s computing cluster (Intel Xeon E5-2683 v4 CPUs and a Tesla T4 GPU) and the University of Cambridge’s CSD3 computing cluster (Tesla P100 PCIe GPUs and Xeon Gold 6142 CPUs). In addition to estimating the carbon footprint, where possible, we provided estimations on how these footprints scale as the inputs vary.

### Estimating the Carbon Footprint

The carbon footprint of a given tool was calculated using the framework described in Lannelongue et al. (2021) and the corresponding online calculator www.green-algorithms.org (last accessed 2022). We present here an overview of the methodology.

Electricity production emits a variety of GHGs, each with a different impact on climate change. To summarize this, the carbon footprint is measured in kilograms of CO_2_-equivalent (CO_2_e), which is the amount of carbon dioxide with an equivalent global warming impact as a mix of GHGs. This indicator depends on two factors: the energy needed to run the algorithm, and the global warming impact of producing such energy, called CI. This can be summarized by:

(1) C = E ×CI,

where *C* is the carbon footprint (in kilograms of CO_2_e—kgCO_2_e), *E* is the energy needed (in W), and CI is the carbon intensity (in kgCO_2_e/W).

The energy needs of an algorithm are measured based on running time, processing cores used, memory deployed, and efficiency of the data center:

(2) $$E\;=\;t\;\times (n_{c}\times P_{c\;}\times u_{c}+n_{m}\times P_{m})\times \mathrm{PUE}\times 0.001,$$

where *t* is the running time (h), *n*_c_ is the number of computing cores, used at *u*_c_%, the core usage factor (between 0 and 1), and each core drawing a power *P*_c_ (W). *n*_m_ is the size of memory available (GB), drawing a power *P*_m_ (W/GB). PUE is the power usage effectiveness of the data center.

The power drawn by a processor (CPU or GPU) is estimated by its thermal design power per core, which is provided by the manufacturer, and then scaled by the core usage factor *u*_c_. The power draw from memory was estimated to be 0.3725 W/GB. The PUE represents how much extra energy is needed to run the computing facilities, mainly for cooling and lighting.

The CI varies between countries because of the heterogeneity in energy production methods, from 0.012 kgCO_2_e/kWh in Switzerland to 0.88 kgCO_2_e/kWh in Australia for example (Carbonfootprint.Com – International Electricity Factors 2020). In order to be location-agnostic in this study, we used the global average value (0.475 kgCO_2_e/kWh; Emissions – Global Energy & CO2 Status Report 2019 – Analysis 2019), unless otherwise specified.

## Supplementary Material

Supplementary data are available at *Molecular Biology and Evolution* online.

## Supplementary Material

msac034_Supplementary_Data
