# Supplementary material for: The Carbon Footprint of Bioinformatics
Source: Mol Biol Evol. 2022 Feb 10;39(3):msac034. doi: 10.1093/molbev/msac034 (PMC8892942; doi:10.1093/molbev/msac034)
Supplement: msac034_Supplementary_Data [file msac034_supplementary_data.zip › Additional file 3.docx]

# Supplementary materials

**Supplementary table 1:** The percentage increase of carbon footprint as a function of memory over-allocation for a given algorithm.

| **Analysis type** | | **Tool** | **Percentage increase in carbon footprint as a function of memory over-allocation (%)** | | | | |
| --- | --- | --- | --- | --- | --- | --- | --- |
|  |  |  | **2x**  **fold** | **5x**  **fold** | **10x fold** | **20x fold** | **50x fold** |
| RNA sequencing quality control pipeline | | FastQC + TrimGalore + clumpify + STARv2.7.0e | 2.50 | 6.25 | 12.49 | 24.99 | 62.47 |
| *De novo* assembly of one human genome | | ABySS2.0 | 2.26 | 5.64 | 11.29 | 22.58 | 56.44 |
|  |  | MEGAHIT | 12.00 | 29.99 | 59.98 | 119.96 | 299.91 |
| Metagenome assembly from 100 soil samples | | MetaSPAdes | 0.33 | 0.84 | 1.67 | 3.35 | 8.37 |
|  |  | MEGAHIT | 0.09 | 0.22 | 0.43 | 0.86 | 2.16 |
|  |  | MetaVelvet k101 | 0.35 | 0.89 | 1.77 | 3.54 | 8.86 |
| GWAS on a biobank with 1 trait | | BOLT-LMM v1 | 45.87 | 114.68 | 229.36 | 458.72 | 1146.81 |
|  |  | BOLT-LMM v2.3 | 45.87 | 114.68 | 229.36 | 458.72 | 1146.80 |
| Read alignment | Human (*Homo sapiens* hg19) | STAR v 2.5.0 | 12.77 | 31.92 | 63.84 | 127.69 | 319.22 |
|  |  | HISAT2 v2.0.0beta | 0.98 | 2.46 | 4.91 | 9.83 | 24.57 |
|  |  | Tophat v2.1.0 | 4.00 | 9.99 | 19.99 | 39.97 | 99.93 |
|  |  | Novoalign | 74.65 | 186.63 | 373.25 | 746.51 | 1866.27 |
|  | Malaria (*Plasmodium falciparum*) | STAR v 2.5.0 | 1.89 | 4.71 | 9.43 | 18.86 | 47.15 |
|  |  | HISAT2 v2.0.0beta | 0.20 | 0.51 | 1.02 | 2.04 | 5.10 |
|  |  | Tophat v2.1.0 | 2.73 | 6.82 | 13.64 | 27.29 | 68.22 |
|  |  | Novoalign | 42.16 | 105.41 | 210.81 | 421.63 | 1054.07 |
| Phylogenetics | Codon modelling | BEAST/  BEAGLE | 8.30 | 20.75 | 41.49 | 82.98 | 207.45 |
|  | Nucleotide modelling |  | 15.55 | 38.87 | 77.74 | 155.47 | 388.68 |
|  | Phylogeographic modelling |  | 15.54 | 38.86 | 77.72 | 155.44 | 388.61 |
| Long read genome Scaffolding | SGA | | 57.61 | 144.03 | 288.05 | 576.10 | 1440.26 |
|  | SSPACE | | 63.70 | 159.24 | 318.49 | 636.97 | 1592.44 |
|  | SOAPdenovo2 | | 56.62 | 141.55 | 283.10 | 566.20 | 1415.50 |
| Short read genome scaffolding | SGA | | 57.73 | 144.32 | 288.64 | 577.29 | 1443.22 |
|  | SSPACE | | 55.05 | 137.62 | 275.24 | 550.47 | 1376.18 |
|  | SOAPdenovo2 | | 56.03 | 140.08 | 280.15 | 560.30 | 1400.76 |
| Transcript isoform abundance estimation | RSEM | | 26.15 | 65.39 | 130.77 | 261.54 | 653.86 |
|  | Sailfish | | 21.41 | 53.52 | 107.04 | 214.07 | 535.18 |
|  | Cufflinks | | 30.48 | 76.20 | 152.40 | 304.79 | 761.98 |
| Metagenomic classification | Centrifuge - short read | | 32.69 | 81.73 | 163.46 | 326.91 | 817.28 |
|  | Kraken2 - short read | | 47.16 | 117.90 | 235.80 | 471.61 | 1179.02 |
|  | Kraken/Bracken - short read | | 99.25 | 248.12 | 496.24 | 992.47 | 2481.18 |
|  | MetaMaps - long read | | 106.65 | 266.62 | 533.24 | 1066.48 | 2666.19 |

##

**Supplementary table 2**: The carbon footprint of hardware changes and parallelisation, using benchmarks from Beale et al [32].

| **Task** | **Algorithm** | **Number of CPU cores or GPU devices** | **Running time (hours)** | **Carbon footprint**  **(kgCO_2_e)** |
| --- | --- | --- | --- | --- |
| **Codon substitution modelling** | BEAST/  BEAGLE | 1  2  4  6  8  10  12 | 7.75  4.17  2.42  1.72  1.42  1.25  1.08 | 0.066  0.069  0.078  0.083  0.091  0.10  0.10 |
|  |  | 1 GPU  2 GPU | 0.08  0.06 | 0.017  0.023 |
| **Nucleotide substitution modelling** | BEAST/  BEAGLE | 2  4  6  8  10  12 | 0.67  0.43  0.40  0.39  0.43  0.43 | 0.012  0.015  0.020  0.026  0.035  0.042 |
|  |  | 1 GPU  2 GPU | 0.27  0.19 | 0.054  0.076 |
| **Phylogeographic modelling** | BEAST/  BEAGLE | 2  4  6  8  10 | 3.86  3.73  3.69  3.71  3.68 | 0.070  0.13  0.18  0.24  0.30 |
|  |  | 1 GPU  2 GPU | 0.64  0.54 | 0.13  0.22 |

**Supplementary Note 1:**

**Estimating the running time at which a GPU has a lower carbon footprint:**

From rearranging the Green Algorithms carbon footprint formula it can be shown that the running time at which GPU has a lower carbon footprint is:

$t_{GPU,eq}=t_{CPU\times}\left( \frac{n_{CPU}\times P_{CPU}\times U_{CPU} +n_{mem,CPU}\times P_{mem}}{n_{GPU}{\times P}_{GPU}\times U_{GPU}+ n_{mem,GPU}\times P_{mem}} \right)$ (1)

Where, $n_{CPU}$is the number of CPU cores, $n_{GPU}$is the number of GPUs, $P_{CPU}$ is the power drawn by the CPU cores. $P_{GPU}$ is the power drawn by the GPU. $U_{CPU}$is the core usage factor for the CPU. $U_{GPU}$ is the usage factor of the GPU. $n_{mem,CPU}$is the amount of memory (GB) utilised when running the CPU, $n_{mem,GPU}$is the amount of memory (GB) utilised when running the GPU. $P_{mem}$is the power draw for memory. $t_{GPU,eq}$is the running time when the GPU would have the same carbon footprint as the CPU, and $t_{CPU}$is the running time of the CPU. If the GPU implementation is to have a lower carbon footprint, it must finish within the time $t_{GPU,eq}$.

When ignoring memory and utilising 1 CPU and 1 GPU with identical core usage factors, this simplifies to:

$$t_{GPU}=t_{CPU\times}\left( \frac{P_{CPU}}{P_{GPU}} \right) (2)$$

Where, $t_{CPU}$ is scaled by the ratio of the power required to utilise the CPU to the GPU.
